# Effect of dexmedetomidine on the median effective concentration of ropivacaine for postoperative analgesia in transversus abdominis plane block: an up-down sequential allocation study

**DOI:** 10.3389/fmed.2025.1491849

**Published:** 2025-04-28

**Authors:** Qin Ye, Hongchun Xu, Xiao Liu, Xujiao Wang, Fangjun Wang

**Affiliations:** ^1^Department of Anesthesiology, Zigong Fourth People's Hospital, Zigong, China; ^2^Affiliated Hospital of North Sichuan Medical College, Nanchong, China

**Keywords:** dexmedetomidine, ropivacaine, transversus abdominis plane block, EC_50_, postoperative pain

## Abstract

**Background:**

This study aimed to observe the effect of dexmedetomidine on the median effective concentration (EC_50_) of ropivacaine for postoperative analgesia in ultrasound-guided transversus abdominis plane block.

**Methods:**

Patients undergoing elective laparoscopic cholecystectomy were randomly divided into the RD group and the R group. In the RD group, 40 mL of ropivacaine with 1 μg/kg dexmedetomidine was injected into the transverse abdominis plane, while subjects in the R group received equal volumes of ropivacaine with normal saline. When the visual analogue scale (VAS) ≤ 3 within 6 h after surgery, postoperative analgesia was assessed as effective. The probit regression was used to calculate the EC_50_ and effective concentration in 95% of patients (EC_95_) of ropivacaine for ultrasound-guided transversus abdominis plane block. The Quality of Recovery-40 (QoR-40) Score on 24 h after surgery and the incidence of adverse reactions were recorded.

**Results:**

The EC_50_ of ropivacaine calculated by the probit regression was 0.207% (95% CI, 0.188% ~ 0.228%) in the R group and 0.165% (95% CI, 0.146% ~ 0.182%) in the RD group. The EC_95_ of ropivacaine was 0.255% (95% CI, 0.230% ~ 0.499%) in the R group and 0.209% (95% CI, 0.187% ~ 0.430%) in the RD group. The score of physical comfort, emotional state, pain, and global score of QoR-40 on 24h after the operation in the RD group was higher than the R group (*p*=0.036, 0.035, 0.027 and 0.020, respectively). There were no significant differences in the incidence of adverse reactions between the two groups.

**Conclusion:**

Dexmedetomidine as a local anesthetic adjuvant can reduce the EC_50_ and EC_95_ of ropivacaine and improve the quality of postoperative recovery of patients with transversus abdominis plane block.

## 1 Introduction

Laparoscopic cholecystectomy is a common surgical procedure. Although it is less invasive than open cholecystectomy, it may lead to moderate-to-severe postoperative pain (1). After laparoscopic cholecystectomy, the most severe and common pain occurred in abdominal wall incisions, which occurred in 41.1% of patients and required analgesics in 73.8% of patients (2). Opioids are commonly used postoperative analgesics, which can effectively relieve postoperative pain. However, opioids have many adverse reactions, such as respiratory depression, nausea and vomiting, gastrointestinal paralysis, and urinary retention, which may outweigh the benefits of analgesia (3).

Transversus abdominis plane (TAP) block was used for postoperative analgesia in patients undergoing cholecystectomy, radical gastrectomy, and hepatectomy (4). The TAP is similar to the intercostal region, which belongs to the high blood perfusion plane. However, a large volume of local anesthetics was usually required to achieve satisfactory analgesic effects, which could cause local anesthetic systemic toxicity (LAST) (5). When ropivacaine was administered at doses of 2.5 mg/kg or 3 mg/kg for the TAP block, the patient’s plasma ropivacaine concentration approached or exceeded the local anesthetic potential toxic concentration (2.2 μg/mL), up to 4 μg/mL (5–7). A patient with renal dysfunction was given 1.8 mg/kg ropivacaine during TAP block; the plasma ropivacaine concentration reached 2.5 μg/mL, and the patient experienced local anesthetic intoxication (8). The ED_50_ (2.05 ~ 3.27 mg/kg) of ropivacaine in TAP blocks in reversal of ileostomy was close to the toxic threshold (3 mg/kg) (9). Therefore, it is necessary to reduce the dose of local anesthetics in TAP blocks to lower the risk of local anesthetic intoxication.

Adding adjuvants can improve the nerve-blocking effect of local anesthetics and reduce the dosage of local anesthetics (10). As a highly selective α_2_ adrenergic receptor agonist, dexmedetomidine combined with local anesthetics has been shown to enhance the analgesic effect of nerve blocks, prolong the action time, and reduce the dosage of postoperative analgesics (11, 12). A meta-analysis also showed the safety and efficacy of dexmedetomidine in combination with local anesthetics for nerve blocks (13). In a previous study (14), Mostafa et al. found that levobupivacaine combined with dexmedetomidine during TAP block in pediatric laparoscopic orchiopexy could attenuate postoperative pain, prolong analgesic time, reduce the dosage of analgesics, and have the highest postoperative satisfaction and minimal sedation. In lumbo-sciatic nerve block and transversal plane block, dexmedetomidine, as a local anesthetic adjuvant, prolonged sensory and motor block time and decreased postoperative pain score in a dose-dependent manner. However, the incidence of adverse reactions such as bradycardia and excessive sedation increased significantly after the dose of dexmedetomidine reached 2 μg/kg (15, 16). Another study (17) suggested that 1 μg/kg dexmedetomidine was recommended as an adjuvant to ropivacaine for TAP block, compared to 0.5 μg/kg and 1.5 μg/kg. Therefore, we chose 1 μg/kg dexmedetomidine as an adjuvant to ropivacaine for TAP block in our study.

However, there has been no report on the effect of dexmedetomidine as an adjuvant on the local anesthetic dosage for postoperative analgesia in the TAP block. We hypothesized that the addition of dexmedetomidine as an adjuvant could reduce the local anesthetic dosage for postoperative analgesia in the TAP block. Therefore, we conducted this study to investigate the effect of 1 μg/kg of dexmedetomidine as a local anesthetic adjuvant on the EC_50_ of ropivacaine for postoperative analgesia in the TAP block.

## 2 Materials and methods

### 2.1 Ethics and patients

This trial was approved by the Medical Ethics Committee of the Affiliated Hospital of North Sichuan Medical College (number 2020ER081-1) and registered in the Chinese Clinical Trial Registry (registration number ChiCTR2000032991, registration date: 17 May 2020). Patients who underwent elective laparoscopic cholecystectomy at the Affiliated Hospital of North Sichuan Medical College were enrolled. All patients signed a written informed consent form. The inclusion criteria were age 18 to 65 years old, American Society of Anesthesiologists (ASA) physical classification status of I–II, and 18 kg/m^2^ ≤ body mass index (BMI) ≤ 30 kg/m^2^. The exclusion criteria included patients with preoperative coagulation abnormalities, skin infection, and breakage at the puncture site, and recent use of sedatives and opioids. The withdrawal criteria included intraoperative massive bleeding, failure of TAP block, a change in the surgical plan, placement of a drainage tube and local anesthetic toxicity, a request from the patient or relative to withdraw from the study, and incomplete data collection.

### 2.2 Randomization and blinding

A computer-generated randomized sequence was used for randomization. Sixty patients who underwent elective laparoscopic cholecystectomy were randomized into two groups: R group with ropivacaine only and the RD group with ropivacaine and 1 μg/kg dexmedetomidine. Prepared 60 cards marked with different numbers (generated by a computer-generated random number) and put the cards in identical and opaque sealed envelopes. When the patient arrived in the operating room, the anesthesia nurse, who did not participate in the trial, randomly selected an envelope and prepared the drugs according to the groups corresponding to the numbers in the envelope. The drugs in both groups were diluted to 40 mL with 0.9% normal saline. The anesthesiologists, patients, statisticians, and data collectors did not know the group allocation. After the study was completed, the data collectors reported the data back to the statisticians. The statisticians analyzed the results and obtained the concentration of ropivacaine for the next patient. When severe local anesthetic toxicity occurred, the patient was unblinded midway and excluded.

### 2.3 Design

Based on the results of previous studies (18, 19) and the preliminary experiment, the concentration of ropivacaine for the first patient in each group was 0.2%. The concentration of ropivacaine for the following patients was determined by the up-down allocation methodology, and the ratio of adjacent concentrations was 1.1 (20). If the analgesia was ineffective, the next patient would be received at a higher concentration. If the analgesia was effective, the next patient would be received with a lower concentration. The effectiveness of analgesia was determined according to the visual analogue scale (VAS) score (0, no pain; 1–3, slight but tolerable pain; 4–6, medium-level pain with groaning and requiring medical treatment; and 7–10, severe completely intolerable pain) (21) within 6 h after surgery. A VAS score ≤ 3 within 6 h after surgery was defined as effective analgesia. If the VAS score > 3 within 6 h after surgery, the postoperative analgesia was ineffective, and 1 mg/kg of tramadol was administered each time for analgesia (22). Pain is an important problem after laparoscopic cholecystectomy, especially the most significant pain in patients within 6 h after surgery (23). When the VAS score was above 3, it affected patient comfort, recovery, potential for same-day discharge, and overall satisfaction (24). Therefore, we set the inflection point as a VAS score > 3 or a VAS score ≤ 3 within 6 h after surgery.

### 2.4 Anesthetic procedure

One day prior to surgery, we thoroughly explained the research protocol to both patients and their families, and familiarized the patients with the VAS and QoR-40 scoring systems. All patients fasted from solid food for 8 h and clear liquids for 4 h before surgery. After entering the anesthesia preparation room, the peripheral vein was accessed, and 10 mL/kg/h Ringer lactate solution was administered intravenously. Electrocardiography (ECG), non-invasive blood pressure (NIBP), and oxygen saturation (SpO_2_) were monitored. Oxygen inhalation through nasal cannula was 2 L/min. After sterilizing the skin, a 5–15 MHz linear high-frequency ultrasound probe was used to perform the subcostal transversus abdominis plane block, which was performed by an experienced anesthesiologist. First, the linear probe was placed below the xiphoid and then paralleled to the costal margin and scanned obliquely along the lower costal margin to identify the rectus abdominis, the transversus abdominis muscle, the internal oblique muscles, and the external oblique muscles. Part of the transversus abdominis muscle extends underneath the rectus abdominis muscle. An 80-mm plexus stimulation needle was used to insert 1 to 2 cm in front of the mark point of the probe with the in-plane technique. When the needle tip was between the rectus abdominis and the transverse abdominis muscles, the aspiration was free of blood and air. Then, 1 ~ 2 mL of the test drug was injected to confirm its location. When a convex lens-shaped dark fluid area appeared between the rectus abdominis and the transversus abdominis muscles, it proved that the needle tip was accurately positioned. The mixed solution of local anesthetic and dexmedetomidine was administered at a speed of 20 mL/30s. All patients underwent bilateral TAP blocks, and 20 mL of the mixed solution was administered to each side. This procedure was completed within 15 min, and then alcohol swabs were used to identify the plane every 5 min. When the cold sensation disappeared or weakened from T_6_ to T_10_ after 20 min of the TAP block, the block was considered successful.

After patients entered the operating room, ECG, NIBP, SpO_2_, end-tidal carbon dioxide partial pressure (PETCO_2_), and bispectral index (BIS) were monitored. The induction of general anesthesia was administered by intravenous propofol (1.5 ~ 2 mg/kg), sufentanil (0.4 μg/kg), and rocuronium (0.6 mg/kg). Then, tracheal intubation was performed, followed by mechanically controlled ventilation. The pure oxygen flow was 2 L/min, the tidal volume was 8 mL/kg, the respiratory rate was 14 times/min, and the inhalation/exhalation ratio was 1:2. Respiratory parameters were adjusted according to PETCO_2_ maintained at 35 ~ 45 mmHg, and SpO_2_ remained above 98%. Intraoperative anesthesia was maintained by intravenous infusion of propofol (4 ~ 8 mg/kg^/^h) and remifentanil (0.1 ~ 0.2 μg/kg/min), which were adjusted according to BIS values (40 ~ 60). Muscle relaxants were added as needed during the operation and discontinued 45 min before the end of the surgery, while propofol and remifentanil were discontinued 5 min before the end of the surgery. When the patient suffers from local anesthetic intoxication, the drug should be stopped immediately, and oxygen should be provided through a mask. Mildly excited patients were given midazolam (0.05 ~ 0.1 mg/kg). Patients who experienced convulsions received intravenous propofol (1 ~ 2 mg/kg). Severe cases were intubated, and vasoactive drugs were used to maintain hemodynamic stability. When the blood pressure decreased by more than 20% of the base value or the systolic blood pressure (SBP) was below 90 mmHg, ephedrine (6 ~ 10 mg) was administered immediately. When the heart rate (HR) was less than 50 beats per minute, atropine 0.5 mg was given. The body temperature of the patients was maintained intraoperatively at approximately 36°C. All patients were positioned with their head elevated at 30° and a leftward inclination of 15°, while abdominal pressure was maintained at 12 ~ 14 mmHg during surgery. Laparoscopic cholecystectomy was performed using a three-port procedure, and all patients underwent the same technique and incision.

After surgery, the patients met the indications for extubation (call for open eyes and tidal volume >5 mL/kg), and then the catheter was extracted and transferred to the post-anesthesia care unit (PACU). If postoperative nausea and vomiting (PONV) required medication, ondansetron 4 mg was administered intravenously. When the patient’s VAS >3, intravenous tramadol 1 mg/kg was administered.

### 2.5 Measurements

The primary outcome of the study included the number of “effective” and “ineffective” responses for each concentration category for each group, which was used to calculate the median effective concentration (EC_50_) and effective concentration in 95% of patients (EC_95_) of ropivacaine. The secondary outcomes included the QoR-40 score of patients 24 h after surgery and adverse reactions. The QoR-40 score includes five parts: physical independence, physical comfort, psychological support, emotional state, and pain. Adverse reactions such as hypotension, bradycardia, postoperative nausea and vomiting, skin itching, and hematoma formation were also recorded. There are four levels of postoperative nausea and vomiting (0 grade: no nausea or vomiting, I grade: nausea but no vomiting, II grade: vomiting once or twice, and III grade: vomiting on more than two occasions) (25).

### 2.6 Statistical analysis

A previous study (20, 26) has shown that an up-down sequential allocation study usually requires 20–40 samples to obtain 6 or more inflection points to calculate the EC_50_ of the drug. The study was conducted with 30 patients in each group with an expected loss of follow-up rate of 10%.

Data analysis was performed using GraphPad Prism version 5.0 and IBM SPSS 23.0 statistical software. Continuous variables with normal distribution were expressed as mean±standard deviation (_X ± SD_), and comparison between groups was performed by two independent samples *t-*tests. Non-normally distributed data were presented as median (interquartile range) M (IQR), and the Mann–Whitney U-test was used for comparison between groups. Categorical data were determined using the *χ*^2^ test or Fisher’s exact test. The EC_50_, EC_95,_ and 95% CI of ropivacaine were calculated according to the probit regression. Overlapping CI methodology, where differences in group means were considered statistically significant when the 83% CIs did not overlap, was also used to test the differences in EC_50_ values between groups as a sensitivity test (20, 26). A *p-*value of <0.05 was considered statistically significant.

## 3 Results

**Figure 1:**
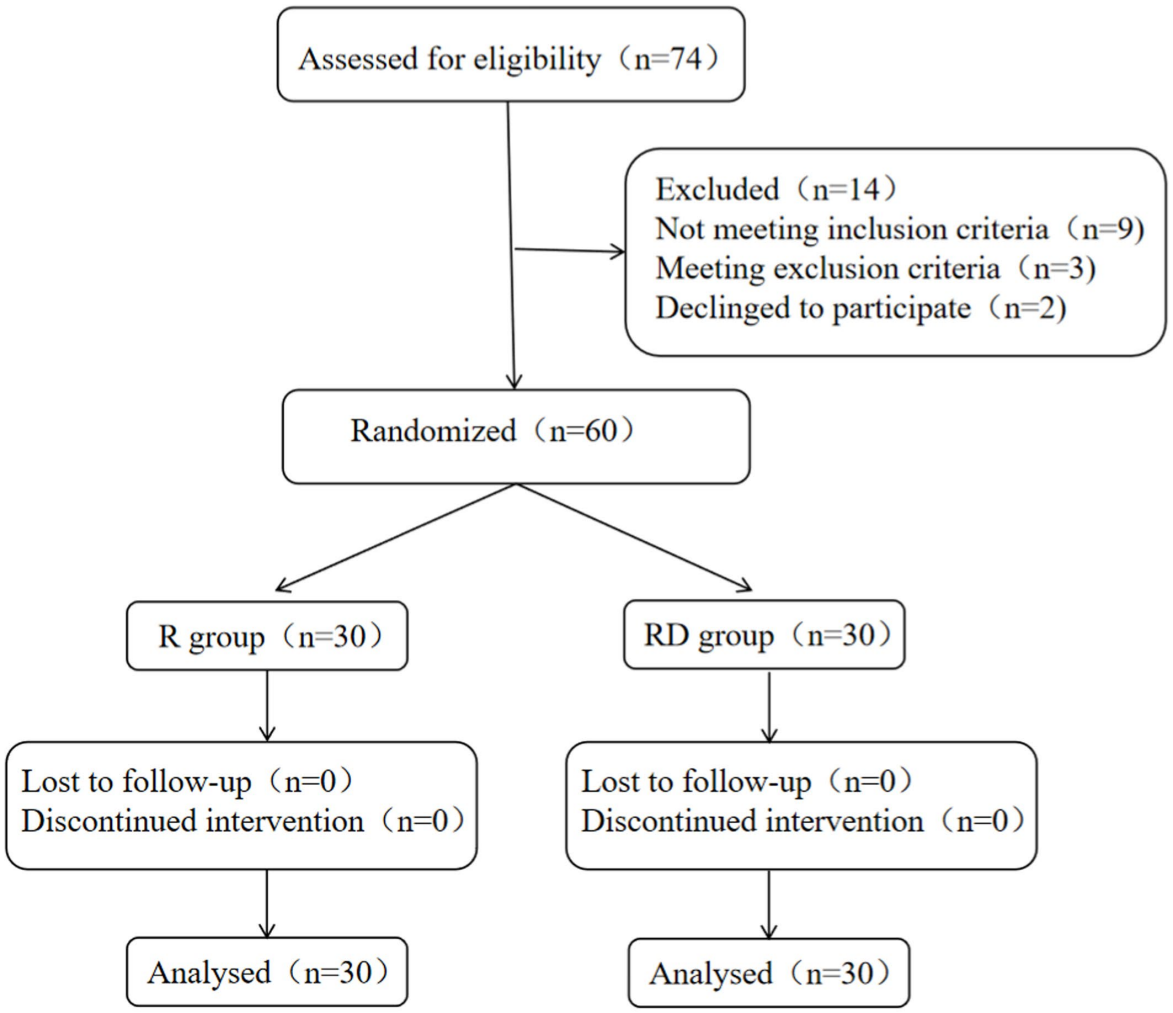
Flow diagram of the study.

A total of 74 patients were recruited for the study; 9 patients did not meet the inclusion criteria, and 3 patients were excluded. In addition, two patients declined to participate. A total of 60 patients completed the study as shown in Figure 1.

There were no significant differences in age, gender, ASA, height, weight, anesthesia time and operation time between the two groups (*p* = 0.796, 0.793, 0.796, 0.385, 0.269, 0.953 and 0.726, respectively) as shown in Table 1.

**Table 1:** Demographic data and patients’ characters.

Variables	R group (*n* = 30)	RD group (*n* = 30)	*t/X^2^* values	*p-*values
Age (years)	47.8 ± 10.7	47.2 ± 8.1	0.259	0.796
Gender				
Male, *n* (%)	12 (40)	13 (43.3)	0.069	0.793
Female, *n* (%)	18 (60)	17 (56.7)		
ASA				
I, *n* (%)	14 (46.7)	15 (50)	0.067	0.796
II, *n* (%)	16 (53.3)	15 (50)		
Height (cm)	161.0 ± 9.1	163.0 ± 8.6	0.875	0.385
Weight (kg)	62.1 ± 11.8	65.5 ± 11.8	1.116	0.269
Duration of anesthesia (min)	67.8 ± 14.3	67.5 ± 16.5	0.059	0.953
Duration of surgery (min)	47.5 ± 14.8	46.1 ± 15.3	0.352	0.726

Data are presented as mean±standard deviation and numbers. ASA, American Society of Anesthesiologists; R group, ropivacaine group; RD group, ropivacaine combined with dexmedetomidine group.

**Figure 2:**
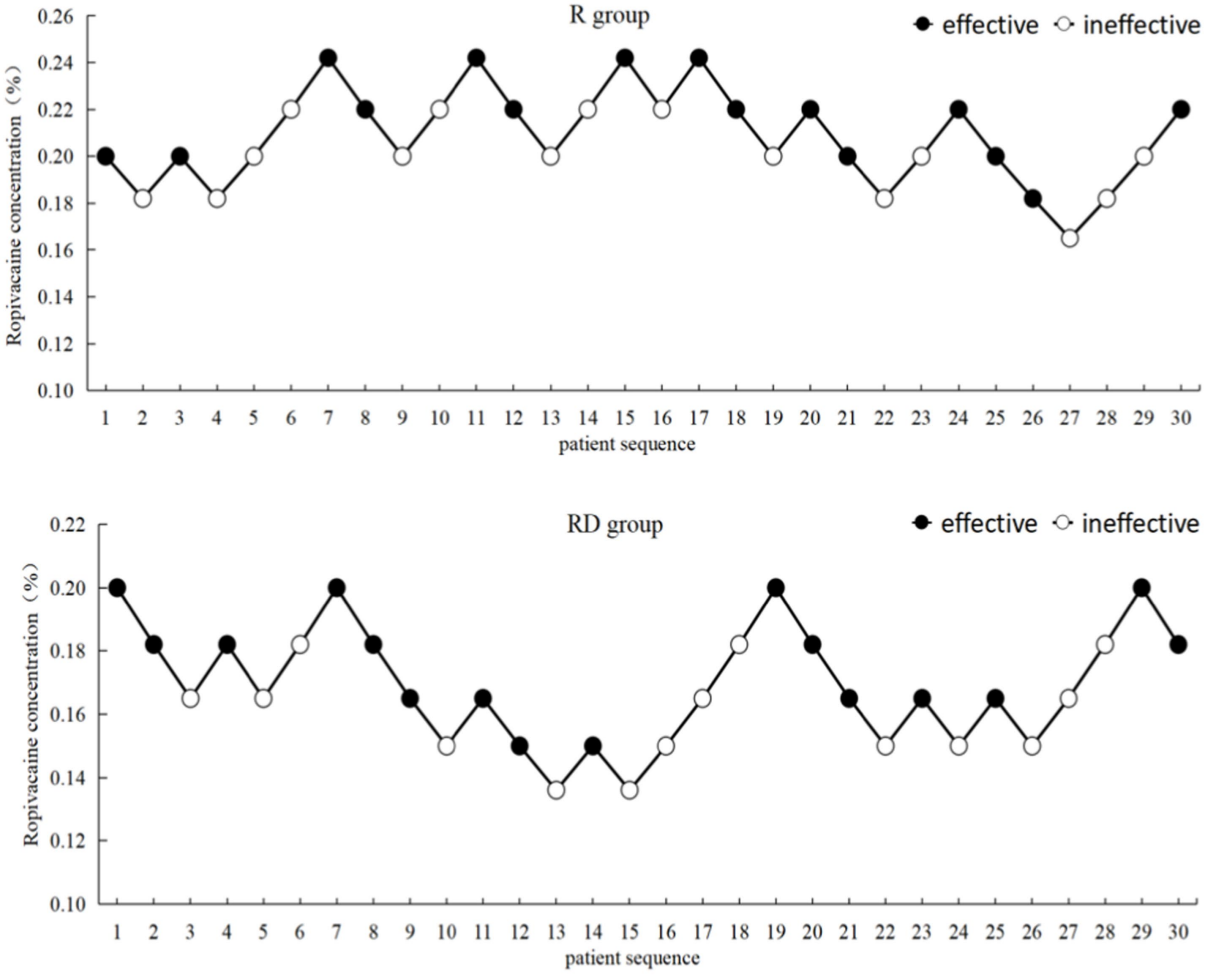
Up-down sequential allocation results.

**Figure 3:**
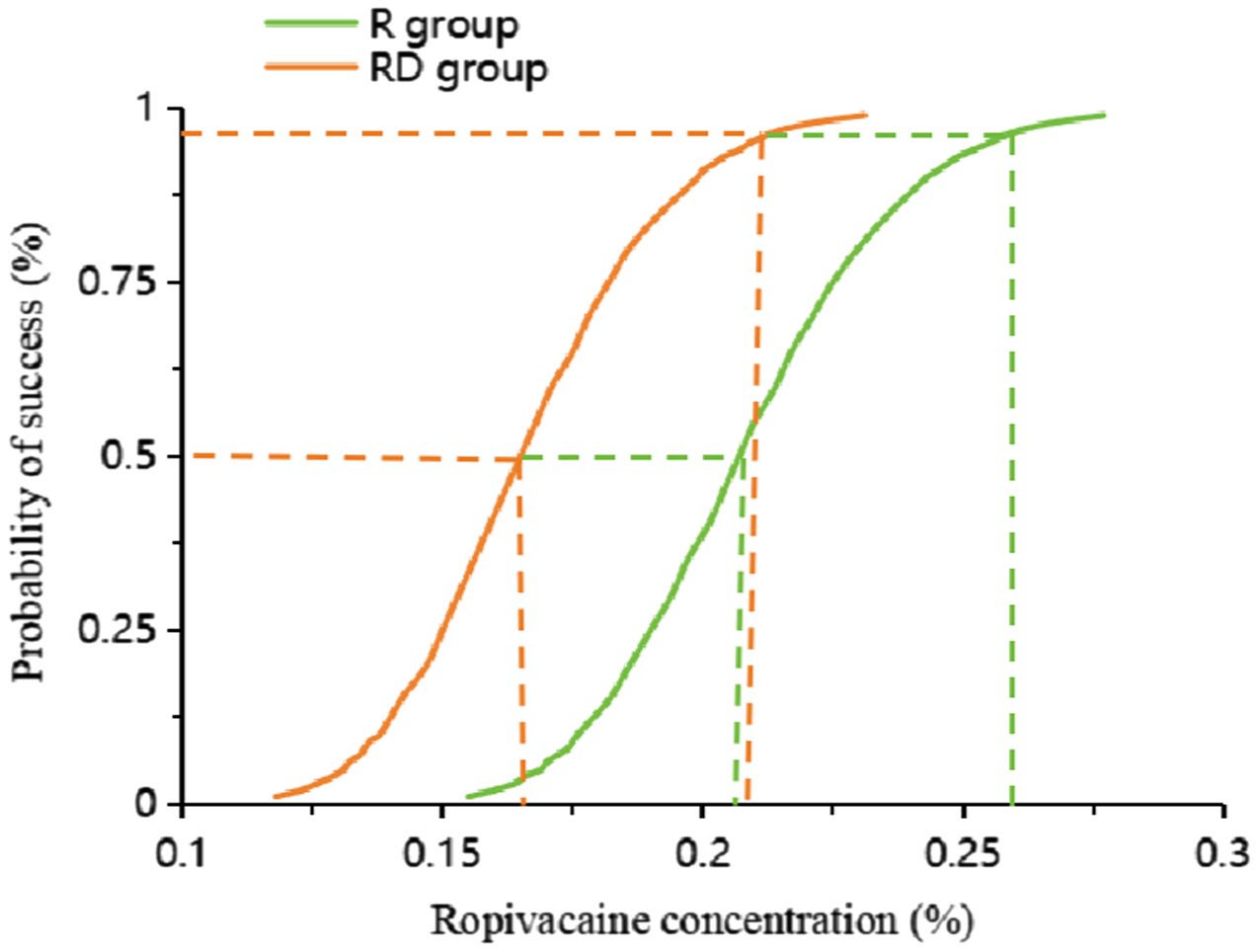
Dose–response curve of the probability of ropivacaine concentration. The values of the EC_50_ and EC_95_ derived from probit analysis were 0.207% (95% CI, 0.188% ~ 0.228%) and 0.255% (95% CI, 0.230% ~ 0.499%) in the R group. The EC_50_ of ropivacaine was 0.165% (95% CI, 0.146% ~ 0.182%), and the EC_95_ was 0.209% (95% CI, 0.187% ~ 0.430%) in the RD group.

The up-down sequential allocation results are shown in Figure 2. According to the probit regression, the EC_50_ of ropivacaine was 0.207% (95% CI, 0.188% ~ 0.228%), and EC_95_ was 0.255% (95% CI, 0.230% ~ 0.499%) in the R group. The EC_50_ of ropivacaine was 0.165% (95% CI, 0.146% ~ 0.182%), and EC_95_ was 0.209% (95% CI, 0.187% ~ 0.430%) in the RD group, as shown in Figure 3.

The physical comfort score, emotional state score, pain score, and total score of the QoR-40 score in the RD group were significantly higher than those in the R group (*p* = 0.036, 0.035, 0.027 and 0.020, respectively), as presented in Table 2. There were no differences between the two groups in the physical independence score and psychological support score of the QoR-40 score (*p* = 1.000 and 1.000), as presented in Table 2.

**Table 2:** QoR-40 score 24 h after surgery.

QoR-40	R group (*n* = 30)	RD group (*n* = 30)	*Z* values	*p*-values
Physical independence, median (IQR)	25 (0)	25 (0)	0.000	1.000
Physical comfort, median (IQR)	52 (6.8)	54 (5.5)*	−2.098	0.036
Psychological support, median (IQR)	35 (0)	35 (0)	0.000	1.000
Emotional state, median (IQR)	41 (4.0)	43 (2.3)*	−2.109	0.035
Pain, median (IQR)	30 (3.5)	32 (4.3)*	−2.202	0.027
Global, median (IQR)	183.5 (9.5)	190 (12.3)*	−2.324	0.020

Data are presented as median (interquartile range). ^*^p < 0.05 vs. R group.

There were no differences in the incidence of hypotension, bradycardia, skin itching, hematoma formation and PONV between the two groups (*p* = 0.103, 0.299, 1.000, 1.000 and 0.434, respectively), as shown in Table 3.

**Table 3:** Adverse reactions in the two groups.

Adverse effects	R group (*n* = 30)	RD group (*n* = 30)	*X^2^* values	*p*-values
Bradycardia, *n* (%)	3 (10)	7 (23.3)	1.920	0.299
Hypotension, *n* (%)	6 (20)	1 (3.3)	4.043	0.103
Skin itching, *n* (%)	0 (0)	0 (0)	0.000	1.000
Hematoma formation, *n* (%)	0 (0)	0 (0)	0.000	1.000
Classification of PONV, *n* (%)				
0	16 (53.3)	16 (53.3)	3.000	0.434
I	2 (6.7)	6 (20)		
II	2 (6.7)	2 (6.7)		
III	10 (33.3)	6 (20)		

Data are presented as numbers. PONV, postoperative nausea and vomiting.

## 4 Discussion

In this up-down sequential allocation study, we found that dexmedetomidine as a local anesthetic adjuvant for TAP block can significantly reduce the EC_50_ and EC_95_ of ropivacaine while improving the quality of postoperative recovery in patients undergoing laparoscopic cholecystectomy.

Laparoscopic cholecystectomy can cause moderate to severe postoperative pain in patients. The TAP block can significantly reduce postoperative pain, lower opioid consumption, and decrease pain scores (27). However, TAP block usually requires a high-volume local anesthetic to dilate the plane and block the nerves that travel through this plane to achieve the analgesic effect. However, the analgesic time of low-concentration local anesthetics is limited for a single TAP block, while high-concentration local anesthetics are associated with the risk of local anesthetic intoxication. Therefore, adding adjuvants to local anesthetics, enhancing the analgesic effect of local anesthetics, and reducing the risk of local anesthetic poisoning have been the focus of research in recent years. Studies (28, 29) have shown that local anesthetic adjuvants fentanyl, sufentanil, clonidine, and dexamethasone can enhance the blocking effect of local anesthesia, but dexmedetomidine has a stronger analgesic effect, which shortens the blocking onset time, prolongs the analgesic time, and reduces postoperative adverse reactions.

In this study, we investigated the EC_50_ and EC_95_ of ropivacaine using the sequential method, which is a simple and scientific approach to assessing the dose–effect relationship of the drug. The EC_50_ and EC_95,_ respectively, represent the concentration of the drug that is effective in 50 and 95% of individuals. In clinical practice, the EC_50_ and EC_95_ of the drug can be used to guide rational drug use. A previous study showed that the ED_50_ (2.05 ~ 3.27 mg/kg) of ropivacaine in TAP blocks in reversal of ileostomy is close to the toxic threshold (3 mg/kg) (9). Therefore, local anesthetic intoxication can easily occur. Raof et al. (30) showed that the EC_50_ of bupivacaine combined with dexmedetomidine was 0.055% for TAP block in children, while the EC_50_ of bupivacaine alone was 0.0839%. In labor epidural analgesia, the EC_50_ of ropivacaine alone was 0.083%, and when ropivacaine was combined with 0.5 ug/ml of dexmedetomidine, the EC_50_ value decreased to 0.062% (31). In these studies, dexmedetomidine was used as an adjuvant for local anesthetics, reducing the EC_50_ of local anesthetics by 34 and 25%, respectively. These are consistent with the results of our study. This study revealed that the EC_50_ of ropivacaine combined with dexmedetomidine for TAP block in adults was 0.165%, whereas the EC_50_ of ropivacaine alone was 0.207%. The combination of ropivacaine with dexmedetomidine resulted in a 20% reduction in EC_50_ compared to ropivacaine alone. A recent study (32) found that the intranasal administration of 1 μg/kg and 2 μg/kg of dexmedetomidine decreased the EC50 of ropivacaine for the caudal block, and there was a specific dose-dependent effect. However, the incidence of hypotension and bradycardia was higher with 2 μg/kg dexmedetomidine as a local anesthetic adjuvant (13, 15, 16). This may be derived from the anti-sympathetic effects of dexmedetomidine, which may inhibit the sympathetic nerve terminal to release norepinephrine and enhance the activity of the vagus nerve (33). There was no difference in the incidence of hypotension or bradycardia between the two groups in our study. However, it is necessary to strengthen the monitoring of the patient’s cardiovascular system to actively prevent and treat complications in clinical applications.

The mechanism by which dexmedetomidine enhances the analgesic effect of local anesthetics has been poorly understood. Current studies have found that dexmedetomidine can inhibit neuronal excitability and A-*α* and C fibers that control pain perception by blocking hyperpolarization-activated cyclic nucleotide-gated (HCN) channels or hyperpolarization-activated cation channel current (*I_h_* current), delay rectifier K^+^ current (*I*_K(DR)_) and Na^+^ current (*I*_Na_) (34–37). The enhancement of sensory and motor blocks with intraneural dexmedetomidine may be related to the anti-inflammatory and neuroprotective properties of dexmedetomidine as a local anesthetic adjuvant. Kim et al. (38) revealed that interleukin (IL)-6 and IL-1β levels, scores of axon, myelinated fiber degeneration, and demyelinated fiber percentages were lower in the ropivacaine plus dexmedetomidine group than in the ropivacaine group. Huang et al. (39) found dexmedetomidine pretreatment elevated brain-derived neurotrophic factor (BDNF) expression by reducing miR-10b-5p expression, thereby alleviating ropivacaine-induced neurotoxicity. Dexmedetomidine can also prevent the absorption of the local anesthetic by vasoconstriction at the injection area and enforce the activity of the peripheral nerve to increase analgesia intensity and duration (40). Meanwhile, the release of peripheral norepinephrine is inhibited, which induces the hyperpolarization of cells and inhibits the transmission of pain signals to the brain center (41). Dexmedetomidine enhances the analgesic effect of anesthetics through these mechanisms. It has also been proven in clinical applications. In brachial plexus block, dexmedetomidine combined with ropivacaine can significantly shorten the onset time of ropivacaine block and extend its block time (42). A meta-analysis (43) showed that local anesthetics combined with dexmedetomidine significantly reduced postoperative pain intensity at 12 h, 24 h, and 48 h, and reduced opioid dosage in femoral nerve block.

The QoR-40 score was used to globally measure the quality of postoperative recovery from five dimensions involving emotional state, physical comfort, psychological support, physical independence, and pain in many clinical trials (44). The results of this study showed that the combination of dexmedetomidine and ropivacaine significantly increased the scores of physical comfort, emotional state, pain, and total score in the 24 h QoR-40 score of patients after surgery more than ropivacaine alone. This suggests that ropivacaine with dexmedetomidine (1 μg/kg) can meet superior pain relief in the early postoperative period, increase patient satisfaction, and improve the quality of postoperative recovery. Similar results were found in Yu Wu et al.’s study of dexmedetomidine combined with ropivacaine was used in deep serratus anterior plane block to improve postoperative recovery quality in patients undergoing modified radical mastectomy (45). Another study (46) discovered that 1 μg/kg (not 0.5 μg/kg) dexmedetomidine combined with ropivacaine in a deep serratus anterior plane block could provide superior postoperative analgesia for patients undergoing modified radical mastectomy. Although a study (47) showed that general anesthesia combined with spinal anesthesia, TAP block, or systemic administration of lidocaine, the quality of recovery on the first day after surgery did not differ from baseline. However, it is not known whether adding dexmedetomidine to the TAP block would have produced different results because clonidine was used in this study. In addition, although local anesthesia in the TAP block was confirmed by ultrasonography to spread at the correct level, the clinical effect could not be evaluated because the block was implemented after the induction of anesthesia.

There are several limitations to our study. First, the plasma concentration of ropivacaine was not detected. However, the concentration of ropivacaine used in this study was low and did not exceed the maximum dose. The median peak venous plasma ropivacaine concentrations were below the reported toxic threshold, even though the concentrations of ropivacaine were up to 0.25% (less than 0.25% in our study) in a previous study (48). Therefore, no patient experienced local anesthesia intoxication during the perioperative period. Second, the sample size of this study is small, and a larger sample study will be needed in the future to confirm the effect of dexmedetomidine on patients’ QoR-40 scores. Third, dexmedetomidine at different doses is used as a local anesthetic adjuvant for TAP block in the clinic, but this study only investigated the effect of 1 μg/kg of dexmedetomidine on the EC_50_ of ropivacaine. We needed to explore whether different doses of dexmedetomidine would have the same effects. Finally, there is a potential bias due to the single-center design and the lack of long-term follow-up.

## 5 Conclusion

In the ultrasound-guided TAP block, the EC_50_ of ropivacaine combined with dexmedetomidine was 0.165% (95% CI, 0.146% ~ 0.182%), and the EC_50_ of ropivacaine alone was 0.207% (95% CI, 0.188% ~ 0.228%). Dexmedetomidine can significantly reduce the EC_50_ of ropivacaine and improve the quality of postoperative recovery for patients undergoing laparoscopic cholecystectomy. Similar results may be achieved in other upper abdominal procedures, such as radical gastrectomy and hepatectomy. However, further research is needed.

## Data Availability

The raw data supporting the conclusions of this article will be made available by the authors, without undue reservation.

## Glossary

EC_50_: the median effective concentration
RD group: 40 mL of ropivacaine with 1 μg/kg dexmedetomidine
R group: 40 mL of ropivacaine with normal saline
VAS: visual analogue scale
95% CI: 95% confidence interval
QoR-40: the Quality of Recovery-40 Score
TAP: transversus abdominis plane
ASA: American Society of Anesthesiologists
BMI: body mass index
ECG: electrocardiography
NIBP: non-invasive blood pressure
SpO_2_: pulse oximetry
PETCO_2_: end-tidal carbon dioxide partial pressure
BIS: bispectral index
SBP: systolic blood pressure
HR: heart rate
PACU: post-anesthesia care unit
PONV: postoperative pain and postoperative nausea and vomiting
M (IQR): median (interquartile range)
$\bar{x}\pm s$: mean±standard deviation
HCN: hyperpolarization-activated cyclic nucleotide-gated
*I_h_* current: hyperpolarization activated cation channel current
*I*_K(DR)_: K^+^ current
*I*_Na_: Na^+^ current
IL: interleukin
BDNF: brain-derived neurotrophic factor
EC_95_: (effective concentration in 95% of patients)
LAST: (local anesthetic systemic toxicity) parameters

## References

[1] JiangBYeS. Pharmacotherapeutic pain management in patients undergoing laparoscopic cholecystectomy: a review. Adv Clin Exp Med. (2022) 31:1275–88. doi: 10.17219/acem/151995, PMID: 36000879

[2] Ure BM Troidl H Spangenberger W Dietrich A Lefering R Neugebauer E. Pain after laparoscopic cholecystectomy. Intensity and localization of pain and analysis of predictors in preoperative symptoms and intraoperative events. Surg Endosc. (1994) 8:90–6. doi: 10.1007/BF00316616, PMID: 8165491

[3] Toleska M Dimitrovski A Shosholcheva M Kartalov A Kuzmanovska B Dimitrovska NT. Pain and multimodal analgesia in laparoscopic cholecystectomy. Pril. (2022) 43:41–9. doi: 10.2478/prilozi-2022-0017, PMID: 35843915

[4] Chen Y Shi K Xia Y Zhang X Papadimos TJ Xu X. Sensory assessment and regression rate of bilateral oblique subcostal transversus abdominis plane block in volunteers. Reg Anesth Pain Med. (2018) 43:174–9. doi: 10.1097/AAP.0000000000000715, PMID: 29278604

[5] Hessian EC Evans BE Woods JA Taylor DJ Kinkel E Bjorksten AR. Plasma ropivacaine concentrations during bilateral transversus abdominis plane infusions. Br J Anaesth. (2013) 111:488–95. doi: 10.1093/bja/aet065, PMID: 23562932

[6] Toju K Shiraishi K Hakozaki T Isosu T Murakawa M. Plasma ropivacaine concentration following ultrasound-guided subcostal transversus abdominis plane block in adults. J Anesth. (2015) 29:146–8. doi: 10.1007/s00540-014-1864-0, PMID: 24935748

[7] Griffiths JD Barron FA Grant S Bjorksten AR Hebbard P Royse CF. Plasma ropivacaine concentrations after ultrasound-guided transversus abdominis plane block. Br J Anaesth. (2010) 105:853–6. doi: 10.1093/bja/aeq255, PMID: 20861094

[8] Ishida T Tanaka S Sakamoto A Hirabayashi T Kawamata M. Plasma ropivacaine concentration after TAP block in a patient with cardiac and renal failure. Local Reg Anesth. (2018) 11:57–60. doi: 10.2147/LRA.S173877, PMID: 30288098 PMC6159791

[9] Lahlou-Casulli M Chaize-Avril C Pouliquen E Desfourneaux V Mazoit JX Malledant Y. The median effective analgesic dose (ED50) of ropivacaine in ultrasound-guided transversus abdominis plane block for analgesia in reversal of ileostomy: a double-blind up-down dose-finding study. Eur J Anaesthesiol. (2015) 32:640–4. doi: 10.1097/EJA.0000000000000198, PMID: 25485878

[10] Schubert AK Wiesmann T Wulf H Obert JDA Eberhart L Volk T. The analgetic effect of adjuvants in local infiltration analgesia - a systematic review with network meta-analysis of randomized trials. J Clin Anesth. (2024) 97:111531. doi: 10.1016/j.jclinane.2024.111531, PMID: 39003958

[11] Modir H Hafez-Alsehe N Almasi-Hashiani A Kamali A. Effects of dexmedetomidine, fentanyl and magnesium sulfate added to ropivacaine on sensory and motor blocks in lower abdominal surgery: a randomized clinical trial. Med Gas Res. (2024) 14:102–7. doi: 10.4103/2045-9912.385947, PMID: 39073337 PMC466983

[12] Bhatia U Khanbhaiwala FB Prajapati N Atodaria A Sutariya V Bamania H. A comparative evaluation of intraperitoneal bupivacaine alone and bupivacaine with dexmedetomidine for post-operative analgesia following laparoscopic cholecystectomy. J Minim Access Surg. (2024) 1:24. doi: 10.4103/jmas.jmas_11_24, PMID: 38958008

[13] Xu Y Kong X. Safety and efficacy of dexmedetomidine in combination with local anesthetics for orthopedic nerve blocks: a systematic review and meta-analysis. Minerva Anestesiol. (2024) 90:427–38. doi: 10.23736/S0375-9393.24.17879-0, PMID: 38771166

[14] Mostafa MF Hamed E Amin AH Herdan R. Dexmedetomidine versus clonidine adjuvants to levobupivacaine for ultrasound-guided transversus abdominis plane block in paediatric laparoscopic orchiopexy: randomized, double-blind study. Eur J Pain. (2021) 25:497–507. doi: 10.1002/ejp.1689, PMID: 33128801

[15] Yu J Shan S Nie Y. Impact of local administration of various doses of dexmedetomidine on ropivacaine-induced lumbar plexus-sciatic nerve block. Exp Ther Med. (2018) 16:711–7. doi: 10.3892/etm.2018.6218, PMID: 30116325 PMC6090230

[16] Zeng Y Wen Y Yang J Sun H. Comparing post-operative analgesic effects of varying doses of dexmedetomidine as an adjuvant to ropivacaine for ultrasound-guided dual transversus abdominis plane block following laparotomy for gynecologic malignancies. Exp Ther Med. (2020) 20:860–7. doi: 10.3892/etm.2020.8787, PMID: 32765653 PMC7388559

[17] Zhang Z Hao D. Optimal dose of dexmedetomidine for preemptive analgesia combined with transversus abdominis plane block after colon cancer surgery. J Nippon Med Sch. (2022) 89:399–404. doi: 10.1272/jnms.JNMS.2022_89-406, PMID: 36031355

[18] Xu C Gu F Wang C Liu Y Chen R Zhou Q. The median effective analgesic concentration of ropivacaine in sciatic nerve block guided by ultrasound after arthroscopic anterior cruciate ligament reconstruction: a double-blind up-down concentration-finding study. Front Med. (2022) 9:830689. doi: 10.3389/fmed.2022.830689, PMID: 35602505 PMC9120939

[19] Alsharari AF Abuadas FH Alnassrallah YS Salihu D. Transversus abdominis plane block as a strategy for effective pain management in patients with pain during laparoscopic cholecystectomy: a systematic review. J Clin Med. (2022) 11:6896. doi: 10.3390/jcm11236896, PMID: 36498471 PMC9735918

[20] Pace NL Stylianou MP. Advances in and limitations of up-and-down methodology: a precis of clinical use, study design, and dose estimation in anesthesia research. Anesthesiology. (2007) 107:144–52. doi: 10.1097/01.anes.0000267514.42592.2a, PMID: 17585226

[21] Xie K Wang Y Teng W He R Li YH Huang SQ. The median effective concentration (EC(50)) of epidural ropivacaine with different doses of oxycodone during limb surgery in elderly patients. Front Med. (2021) 8:808850. doi: 10.3389/fmed.2021.808850, PMID: 35127764 PMC8814631

[22] Korkusuz M Basaran B Et T Bilge A Yarimoglu R Yildirim H. Bilateral external oblique intercostal plane block (EOIPB) in patients undergoing laparoscopic cholecystectomy: a randomized controlled trial. Saudi Med J. (2023) 44:1037–46. doi: 10.15537/smj.2023.44.10.20230350, PMID: 37777270 PMC10541983

[23] Emile SH Elfeki H Elbahrawy K Sakr A Shalaby M. Ultrasound-guided versus laparoscopic-guided subcostal transversus abdominis plane (TAP) block versus no TAP block in laparoscopic cholecystectomy; a randomized double-blind controlled trial. Int J Surg. (2022) 101:106639. doi: 10.1016/j.ijsu.2022.106639, PMID: 35487422

[24] Bourgeois C Oyaert L Van de Velde M. Pain management after laparoscopic cholecystectomy: a systematic review and procedure-specific postoperative pain management (PROSPECT) recommendations. Eur J Anaesthesiol. (2024) 41:841–55. doi: 10.1097/EJA.0000000000002047, PMID: 39129451

[25] Oh AY Kim JH Hwang JW do SH Jeon YT. Incidence of postoperative nausea and vomiting after paediatric strabismus surgery with sevoflurane or remifentanil-sevoflurane. Br J Anaesth. (2010) 104:756–60. doi: 10.1093/bja/aeq091, PMID: 20418533

[26] Liu L Drzymalski D Xu W Zhang W Wang L Xiao F. Dose dependent reduction in median effective concentration (EC (50)) of ropivacaine with adjuvant dexmedetomidine in labor epidural analgesia: an up-down sequential allocation study. J Clin Anesth. (2021) 68:110115. doi: 10.1016/j.jclinane.2020.110115, PMID: 33142249

[27] Park S Park JH Park S Jang JN Kim C Choi YS. Ultrasound-guided subcostal approach of transversus abdominis plane block compared with wound infiltration for postoperative analgesia following laparoscopic cholecystectomy: a systematic review and meta-analysis. Medicine. (2024) 103:e38044. doi: 10.1097/MD.0000000000038044, PMID: 38701299 PMC11062739

[28] Luo J Duan G Huang H Chen G. Research status of different adjuvants on nerve block's effect. Pain Physician. (2024) 27:507–19. doi: 10.36076/ppj.2024.7.507, PMID: 39621975

[29] Yang J Zhao M Zhang X. Ropivacaine with dexmedetomidine or dexamethasone in a thoracic paravertebral nerve block combined with an erector spinae plane block for thoracoscopic lobectomy analgesia: a randomized controlled trial. Drug Des Devel Ther. (2022) 16:1561–71. doi: 10.2147/DDDT.S366428, PMID: 35655534 PMC9152436

[30] Raof RA El MS Alia DA. Dexmedetomidine decreases the required amount of bupivacaine for ultrasound-guided transversus abdominis plane block in pediatrics patients: a randomized study. J Clin Anesth. (2017) 37:55–60. doi: 10.1016/j.jclinane.2016.10.041, PMID: 28235529

[31] Zhang W Li C. EC_50_ of epidural ropivacaine combined with dexmedetomidine for labor analgesia. Clin J Pain. (2018) 34:950–3. doi: 10.1097/AJP.0000000000000613, PMID: 29595529

[32] Wang F Qu S Chen Y Liao B Ao L Zhang H. A randomized double-blinded study assessing the effect of different doses of transnasal dexmedetomidine on the median effective concentration of ropivacaine for a caudal block. Front Med. (2024) 11:1481938. doi: 10.3389/fmed.2024.1481938, PMID: 39624036 PMC11608987

[33] Penttila J Helminen A Anttila M. Cardiovascular and parasympathetic effects of dexmedetomidine in healthy subjects. Can J Physiol Pharmacol. (2004) 82:359–62. doi: 10.1139/y04-028, PMID: 15213737

[34] Zhang T Liao X Chen Y Shu X Liu D Yao Y. Dexmedetomidine prolongs lidocaine intravenous regional anesthesia in rats by blocking the hyperpolarization-activated cation current. Drug Des Devel Ther. (2024) 18:1103–14. doi: 10.2147/DDDT.S450971, PMID: 38618283 PMC11015855

[35] Brummett CM Hong EK Janda AM Amodeo FS Lydic R. Perineural dexmedetomidine added to ropivacaine for sciatic nerve block in rats prolongs the duration of analgesia by blocking the hyperpolarization-activated cation current. Anesthesiology. (2011) 115:836–43. doi: 10.1097/ALN.0b013e318221fcc9, PMID: 21666435 PMC3179561

[36] Chen BS Peng H Wu SN. Dexmedetomidine, an alpha2-adrenergic agonist, inhibits neuronal delayed-rectifier potassium current and sodium current. Br J Anaesth. (2009) 103:244–54. doi: 10.1093/bja/aep107, PMID: 19542547

[37] Butterworth JT Strichartz GR. The alpha 2-adrenergic agonists clonidine and guanfacine produce tonic and phasic block of conduction in rat sciatic nerve fibers. Anesth Analg. (1993) 76:295–301. PMID: 8093828

[38] Kim B Choi J Baek S Lee DH. Effects of intraneural injection of dexmedetomidine in combination with ropivacaine in rat sciatic nerve block. Reg Anesth Pain Med. (2018) 43:378–84. doi: 10.1097/AAP.0000000000000745, PMID: 29505435

[39] Xu W Li X Chen L Luo X Shen S Wang J. Dexmedetomidine pretreatment alleviates ropivacaine-induced neurotoxicity via the miR-10b-5p/BDNF axis. BMC Anesthesiol. (2022) 22:304. doi: 10.1186/s12871-022-01810-6, PMID: 36163004 PMC9511747

[40] Vahedi Z Moshari A Moshari M. Efficacy of adding dexmedetomidine to lidocaine to enhance inferior alveolar nerve block in patients with asymptomatic irreversible pulpitis: double-blind randomized clinical trial. Clin Oral Investig. (2022) 26:4727–34. doi: 10.1007/s00784-022-04436-7, PMID: 35267097

[41] Tang C Xia Z. Dexmedetomidine in perioperative acute pain management: a non-opioid adjuvant analgesic. J Pain Res. (2017) 10:1899–904. doi: 10.2147/JPR.S139387, PMID: 28860845 PMC5565238

[42] Dai W Tang M He K. The effect and safety of dexmedetomidine added to ropivacaine in brachial plexus block: a meta-analysis of randomized controlled trials. Medicine. (2018) 97:e12573. doi: 10.1097/MD.0000000000012573, PMID: 30313043 PMC6203584

[43] Zhao ZF Du L Wang DX. Effects of dexmedetomidine as a perineural adjuvant for femoral nerve block: a systematic review and meta-analysis. PLoS One. (2020) 15:e0240561. doi: 10.1371/journal.pone.0240561, PMID: 33075089 PMC7571703

[44] Miao M Xu Y Li B Chang E Zhang L Zhang J. Intravenous administration of dexmedetomidine and quality of recovery after elective surgery in adult patients: a meta-analysis of randomized controlled trials. J Clin Anesth. (2020) 65:109849. doi: 10.1016/j.jclinane.2020.109849, PMID: 32403055

[45] Wu Y Kang Y Li Y Fu B. Impact of ultrasound-guided deep serratus anterior plane block combined with dexmedetomidine as an adjuvant to ropivacaine inpatient quality of recovery scores undergoing modified radical mastectomy: a randomized controlled trial. Front Oncol. (2022) 12:858030. doi: 10.3389/fonc.2022.858030, PMID: 35433468 PMC9008730

[46] Xu X Chen X Zhu W Zhao J Liu Y Duan C. Efficacy and safety of ultrasound guided-deep serratus anterior plane blockade with different doses of dexmedetomidine for women undergoing modified radical mastectomy: a randomized controlled trial. Front Med. (2022) 9:819239. doi: 10.3389/fmed.2022.819239, PMID: 35198576 PMC8860248

[47] Beilstein CM Huber M Furrer MA Löffel LM Wuethrich PY Engel D. Impact of analgesic techniques on early quality of recovery after prostatectomy: a 3-arm, randomized trial. Eur J Pain. (2022) 26:1990–2002. doi: 10.1002/ejp.2020, PMID: 35960649 PMC9541353

[48] Li Z Tang XH Li Q Zhang WJ Tao T Zhu T. Ultrasound-guided oblique sub-costal transversus abdominis plane block as the principal anesthesia technique in peritoneal dialysis catheter implantation and plasma ropivacaine concentration evaluation in ESRD patients: a prospective, randomized, double-blinded, controlled trial. Perit Dial Int. (2018) 38:192–9. doi: 10.3747/pdi.2017.00222, PMID: 29848599

